# Prognostic significance of receptor conversion following neoadjuvant therapy in breast cancer: a systematic review & meta-analysis

**DOI:** 10.1016/j.breast.2025.104516

**Published:** 2025-06-09

**Authors:** Gavin P. Dowling, Gordon R. Daly, Cian M. Hehir, Maen M. AlRawasdeh, Gavin G. Calpin, Sami Almasri, Sinead Toomey, Leonie S. Young, Bryan T. Hennessey, Arnold D.K. Hill

**Affiliations:** a Department of Surgery, Royal College of Surgeons in Ireland (RCSI), University of Medicine and Health Sciences, Dublin, Ireland; b Medical Oncology Lab, Department of Molecular Medicine, RCSI University of Medicine and Health Sciences, Dublin, Ireland; c Department of Surgery, Beaumont Hospital, Dublin, Ireland

**Keywords:** Breast cancer, Receptor conversion, HER2, Hormone receptor, Neoadjuvant therapy, Prognosis, Targeted therapy

## Abstract

**Purpose:**

Receptor conversion following neoadjuvant therapy in breast cancer may influence prognosis and adjuvant treatment decisions. This systematic review and meta-analysis evaluated the prognostic significance of changes in hormone receptor (HR) and HER2 status after neoadjuvant therapy.

**Methods:**

This study was performed in accordance with PRISMA guidelines. A systematic search of the literature was conducted to identify studies assessing the prognostic effect of receptor conversion after neoadjuvant treatment in breast cancer. Studies reporting receptor status before and after neoadjuvant therapy, with associated survival outcomes, were included. Pooled hazard ratios (HRs) for disease-free survival (DFS) and overall survival (OS) were calculated using random-effects models.

**Results:**

Twenty-two studies (n = 5370) were included in this meta-analysis. HR gain demonstrated significantly improved DFS (HR 0.49, 95 % CI 0.25–0.97; *p* = 0.04), but no OS benefit. HR loss was associated with both significantly worse DFS (HR 3.42, 95 % CI 1.93–6.08; *p* < 0.001) and OS (HR 1.99, 95 % CI 1.04–3.84; *p* = 0.04). HER2 gain had a negative impact on DFS (HR 1.89, 95 % CI 1.00–3.58; *p* = 0.05), with no significant effect on OS. HER2 loss was associated with significantly poorer DFS (HR 1.92, 95 % CI 1.51–2.43; *p* < 0.001) and OS (HR 2.20, 95 % CI 1.44–3.38; *p* < 0.001).

**Conclusion:**

This systematic review and meta-analysis demonstrates that receptor conversion following neoadjuvant therapy in breast cancer significantly impacts survival outcomes. Specifically, gaining HR positivity is associated with improved DFS, while losing HR positivity correlates with worse DFS and OS. With regards to HER2, gaining positivity is associated with worse DFS, and losing positivity is associated with worse DFS and OS, compared to patients who maintain their initial status. These findings underscore the potential importance of reassessing receptor status after neoadjuvant therapy to tailor subsequent treatment decisions accurately.

## Highlights

• Receptor conversion after neoadjuvant therapy significantly impact prognosis in breast cancer.
• HR loss is linked to worse DFS and OS, HR gain improves DFS.
• HER2 loss worsens both DFS and OS, HER2 gain worsens DFS only.
• Findings may support personalised adjuvant treatment based on receptor changes.

## 1 Introduction

The expression of human epidermal growth factor receptor-2 (HER2) and hormone receptors (HR) critically influence breast tumour behaviour and clinical management. It is well established that the expression levels of these receptors are dynamic and may shift in response to treatment [1]. Neoadjuvant therapy is widely recognised as an effective treatment option for locally advanced breast cancer of any subtype, as well as early-stage HER2-positive (HER2+) and triple-negative breast cancer (TNBC). The benefits include downstaging disease and potentially reducing the extent of breast and axillary surgery, and that response to treatment serves as a prognostic indicator [2]. Although there have been significant improvements in tailored regimens in recent years, many patients who receive neoadjuvant treatment have residual disease at surgery, which may guide adjuvant treatment decisions [3,4]. There is substantial evidence that discordance exists between the expression of receptors in the primary tumour compared to the residual disease specimen. This phenomenon has been reported at varying frequencies, with one study reporting changes in HR and HER2 status in 33 % and 15 %, respectively [5].

Receptor conversion is clinically significant because it may affect the choice of adjuvant therapy and potentially the patient's prognosis. For example, the KATHERINE trial demonstrated the importance of assessing tumour response post-neoadjuvant therapy in HER2+ patients, with adjuvant trastuzumab-emtansine (T-DM1) improving outcomes in those with residual disease [4]. However, a sub-analysis showed that even patients with HER2-negative residual disease on retesting benefited from T-DM1, suggesting that treatment decisions can be complex and may not always align with retested status [6]. While trials such as this may indirectly support the need for repeat biomarker testing, there is insufficient evidence that routine retesting after neoadjuvant therapy confers a definite benefit. This is reflected in the lack of guidelines universally recommending repeat receptor testing. The European Society of Medical Oncology (ESMO) guidelines recommends retesting in certain scenarios only; if not previously assessed or if there is a change in tumour grade or type [7]. In addition, neither the American Society of Clinical Oncology (ASCO) and National Comprehensive Cancer Network (NCCN) guidelines specifically address retesting on residual disease, instead relying on initial biomarker testing to guide treatment decisions [8,9].

Despite not being explicitly recommended by guidelines, in practice pathologists often retest receptors on surgical specimens as part of routine assessment when residual disease is present [10]. As a result, there is evidence that this practice may have a clinical impact by altering adjuvant therapy regimens for certain patients [11]. There is also considerable evidence that alterations in receptor expression after neoadjuvant therapy has prognostic implications, although the literature varies on the extent of this [[12], [13], [14]]. A previous meta-analysis evaluated the impact of receptor status conversion, finding that a loss of HR status negatively impacted both disease-free (DFS) and overall survival (OS). However, the limited number of studies included in this analysis restricts our ability to draw definitive conclusions, particularly with regards to HER2 status changes [15].

Therefore, in the era of personalised medicine, this systematic review and meta-analysis aims to elucidate the prognostic impact of receptor conversion following neoadjuvant therapy in breast cancer, with the potential to inform future guidelines.

## 2 Methods

A systematic review and meta-analysis were performed in accordance with PRISMA [16] and MOOSE [17] guidelines. All authors contributed to formulating the study protocol which was registered with the International Prospective Register of Systematic Reviews (PROSPERO registration number CRD420250608960) [18]. Three researchers designed the literature search, retrieved relevant abstracts and full manuscripts, appraised selected studies, and analyzed relevant data.

### 2.1 Inclusion and exclusion criteria

Studies eligible for inclusion were: available full English language papers investigating the prognostic effect of receptor conversion in breast cancer patients undergoing neoadjuvant therapy. To be included in the review, studies must only include patients with breast cancer or, when several cancers were being studied, have identifiable outcomes for the breast cancer sub-cohort. Studies also had to meet the following inclusion criteria: (1) participants received neoadjuvant treatment for breast cancer; (2) reported HR and/or HER2 status before and after neoadjuvant therapy; (3) documented effect of HR and/or HER2 conversion on survival outcomes. Studies were excluded from the analysis if: (1) participants did not receive neoadjuvant treatment for breast cancer; (2) incomplete recording of breast cancer receptor status either before or after neoadjuvant therapy.

### 2.2 Study selection

A systematic review of PUBMED, Embase, the Cochrane Library, and Scopus was performed. Any study published before April 2024 was eligible for inclusion. Combinations of Medical Subject Heading (MeSH) terms and keywords, as well as Boolean operators ‘and’ and ‘or’ were used. Search terms used were: ‘breast neoplasms’, ‘neoadjuvant therapy’, ‘receptor’, ‘molecular subtype’, ‘conversion’, ‘discordance’ and ‘change’. Additionally, the reference lists of manuscripts included were searched for further eligible studies. Titles and abstracts were initially reviewed for suitability and duplicates were manually removed. Full texts of the remaining articles were then reviewed. Assessment of selected articles regarding inclusion criteria was undertaken independently by two authors and subsequently verified by senior authors. Where two papers by the same author appeared to use the same cohort, the more recent paper with the larger cohort and longer follow-up was retained.

### 2.3 Data collection and assessment of quality

Two investigators (G.P.D & G.R.D) independently extracted the following data: study design, number of patients, pre-neoadjuvant biopsy method, HER2 detection method, survival outcome, median follow up, neoadjuvant regimen and hazard ratios, where available. Lead authors were contacted by e-mail and a request for further unpublished data was made when relevant data were not available in original articles. The STROBE checklist [19] and Newcastle-Ottawa scale [20] were used to assess the quality of eligible studies; scores are included in Table S1 and Table S2.

### 2.4 Statistical analysis

A meta-analysis was undertaken of the studies for each of the receptor conversions after neoadjuvant therapy: HR gain, HR loss, HER2 gain and HER2 loss. The hazard ratios (HRs) for survival outcomes (OS or DFS) and the corresponding 95 % confidence intervals (CIs) were extracted from multivariable analyses. In studies where multivariable analysis results were not reported, univariable analysis was taken instead. In studies which had inappropriate references for HRs, they were converted to ensure consistency across comparisons [21]. In studies which did not report HRs, but included Kaplan-Meier survival curves, the data was extracted from graphs using WebPlotDigitaliser (V 5.1), and the extracted data was used to calculate HRs using the spreadsheet designed by Tierney et al. [22,23]. This tool has been shown to have high levels of intercoder reliability and validity [24] In cases where survival curves could not be accurately extracted, they were excluded.

Individual study results and pooled estimates were displayed in forest plots. Heterogeneity was investigated by visual inspection of forest plots and calculation of the *I*^*2*^ statistic, which provides the percentage variability attributed to heterogeneity rather than sampling error. A random-effects model was used in all analyses to account for heterogeneity in the methodological differences in data extraction methods [25]. A *P*-value of ≤0.05 was considered statistically significant. To assess the influence of studies where HRs were derived from digitized Kaplan-Meier curves, a sensitivity analysis was performed by subgrouping studies based on HR derivation method. The pooled effect estimates were consistent between subgroups, and the test for subgroup differences was non-significant, indicating that the inclusion of these studies did not materially affect the results (Supplementary Fig. S1–S4). Statistical analysis was performed using Review Manager (Revman) version 5.3 (*Nordic Cochrane Centre, Copenhagen, Denmark*).

## 3 Results

### 3.1 Literature search

**Fig. 1:**
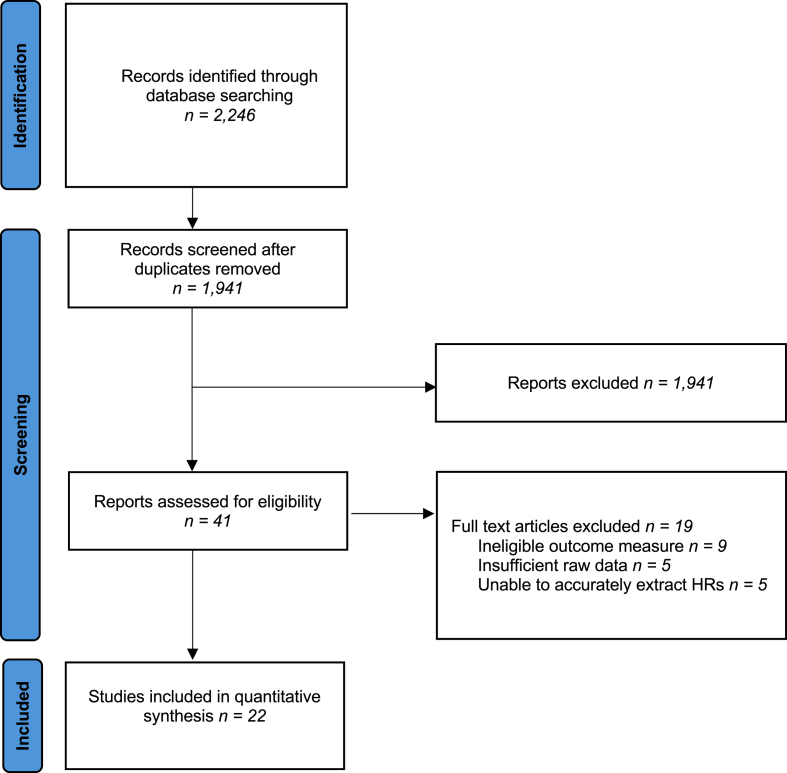
Flow diagram.

The initial search strategy yielded 2246 articles, of which 305 duplicate studies were manually removed. The remaining 1941 titles and abstracts were screened for relevance, of which 41 studies had their full texts assessed for eligibility. Accurate hazard ratios could not be extracted from 6 articles, and were therefore excluded. This resulted in a total of 22 studies included in the meta-analysis, after application of inclusion and exclusion criteria (Fig. 1.).

### 3.2 Study characteristics

Individual study characteristics are reported in Table 1. A total of 5370 patients were included across 22 studies. The reported median follow ups ranged from 1.8 to 6.2 years. Immunohistochemistry (IHC) was used to determine HR status in all studies. IHC ± FISH testing was performed for HER2 status. The majority of studies included neoadjuvant anti-HER2 therapy for HER2+ patients, and adjuvant endocrine therapy for HR-positive (HR+) patients (Table 1). Only one study included patients who received neoadjuvant endocrine therapy. The majority of studies adhered to the ASCO/CAP guideline of defining HR positivity as ≥1 % of tumour nuclei staining for ER/PR on IHC, with only two studies using higher thresholds of ≥10 % [26] and ≥5 % [27] (Supplementary Table S3). Although not reported in all studies, pooling of raw data, where available, demonstrated HR change in 34.1 % of patients (18.1 % gain, 16 % loss) and HER2 change in 12.9 % of patients (4.8 % gain, 8.1 % loss).

**Table 1:** Study characteristics.

Author	Year	Country	Type of study	Number of patients	Patients retested	HER2 Detection	Outcomes	Median follow up (years)	Neoadjuvant regimen	Neoadjuvant endocrine therapy for HR+	Adjuvant endocrine therapy for HR+	Neoadjuvant anti-HER2 therapy for HER2+	Adjuvant anti-HER2 therapy for HER2+
Tacca [26]	2007	France	Retrospective	710	420	IHC	OS, DFS	–	Various regimens	No	Yes (370 patients)	No	–
Mittendorf [63]	2009	USA, Spain	Retrospective	142	25	IHC/FISH	DFS	3.1	Paclitaxel + FEC + Trastuzumab	No	Yes	Yes (all trastuzumab)	Yes
Chen [32]	2012	China	Retrospective	259	224	–	OS, DFS	5	Various regimens	No	Yes	No	No
Guarneri [64]	2013	Italy	Retrospective	107	69	IHC/FISH	DFS	–	Chemotherapy alone or Chemotherapy + trastuzumab/lapatinib	No	Yes	Some (one cohort)	Yes
Tan [28]	2014	China	Retrospective	267	229	IHC	OS, DFS	3.5	Various regimens	No	Yes	No	–
Jin [14]	2015	China	Prospective	423	423	IHC/FISH	OS, DFS	3.7	Various regimens	No	Yes	No	Yes
Parinyanitikul [27]	2015	USA	Retrospective	398	398	IHC/FISH	OS, DFS	3.3	Anthracycline-based, taxane-based, or combination	No	Yes	Some (35 patients – trastuzumab)	Yes
Lim [29]	2016	Korea	Retrospective	322	290	IHC/FISH	OS, DFS	5.3	Various regimens	No	Yes	Some (21 patients – trastuzumab/lapatinib)	Some (21 patients)
Wang [65]	2017	China	Prospective	549	298	IHC/FISH	OS, DFS	3	Paclitaxel + Carboplatin ± Trastuzumab	No	Yes	Some (379 patients – trastuzumab)	Yes
Yoshida [33]	2017	Japan	Retrospective	588	588	IHC/FISH	DFS	6.2	Anthracycline-based, Taxane-based, or combination ± Trastuzumab	No	–	Some (38 patients – trastuzumab)	Some (57 patients)
Branco [66]	2019	Portugal	Retrospective	108	60	IHC/FISH	OS, DFS	4.3	Anthracycline-taxane based chemotherapy ± trastuzumab ± pertuzumab	No	–	Some (87 patients – trastuzumab, 3 patients – trastuzumab + pertuzumab)	Yes
Ignatov [67]	2019	Germany	Retrospective	227	227	IHC/FISH	OS, DFS	–	HER2-targeted therapy	No	–	Some (30 patients – trastuzumab)	Yes
Tural [68]	2019	Turkey	Retrospective	186	151	IHC/FISH	DFS	2.9	Anthracycline-taxane based chemotherapy ± trastuzumab	No	Yes	All (79 patients – trastuzumab)	Yes
Zhao [34]	2019	China	Retrospective	119	119	IHC/FISH	OS, DFS	–	Various regimens	No	Yes	Yes (trastuzumab)	Yes
Khalid Al-Saleh [35]	2021	Saudi Arabia	Retrospective	91	91	IHC/FISH	OS, DFS	–	Various regimens	No	Yes	Yes (trastuzumab)	Yes
Mohan [31]	2021	USA	Retrospective	303	186	IHC/FISH	OS, DFS	5.48	Various regimens	Some (14 patients)	Yes	Yes	Yes
Taha [30]	2021	Egypt	Prospective	110	110	IHC/FISH	OS, DFS	2.3	Various regimens	No	Yes	Some (31 patients – trastuzumab)	Yes
Wetzel [69]	2021	USA	Retrospective	348	121	IHC/FISH	OS, DFS	3.7	Various trastuzumab-based regimens	No	Yes	Yes (trastuzumab or trastuzumab + pertuzumeb)	Yes
Chen [36]	2022	China	Retrospective	670	670	IHC/FISH	OS, DFS	4.3	Various regimens	No	Yes	Some (25 patients – trastuzumab)	Yes
He [5]	2023	China	Retrospective	294	294	IHC/FISH	OS, DFS	6	Various regimens	No	Yes	Some (13 patients – trastuzumab)	–
LeVee [70]	2023	USA	Retrospective	163	102	IHC/FISH	DFS	3.3	Chemotherapy + Dual anti-HER2 therapy	No	–	All (trastuzumab + pertuzumab)	Yes
Ren [71]	2023	China	Retrospective	499	499	IHC/FISH	DFS	1.8	Various regimens + anti-HER2 therapy	No	Yes	Yes (201 patients – trastuzumab, 298 patients – trastuzumab + pertuzumab)	Yes

Abbreviations: IHC; immunohistochemistry, FISH; fluorescence in situ hybridization, OS; overall survival, DFS; disease-free survival, FEC; 5-FU, epirubicin, cyclophosphamide.

### 3.3 Prognostic value of HR gain

**Fig. 2:**
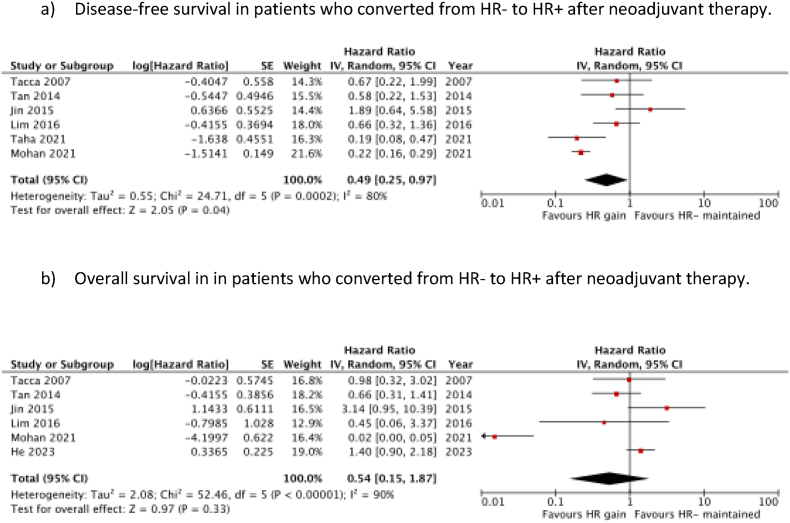
Prognostic significance of HR gain after neoadjuvant therapy.

A total of 6 studies reported the prognostic value of HR gain on DFS [14,26,[28], [29], [30], [31]]. Meta-analysis of these studies (1658 patients) demonstrated a significant association between HR gain and improved DFS (HR 0.49 (95 % c.i. 0.25 to 0.97); *P* = 0.04), in comparison with patients who remained HR-negative (HR-) after neoadjuvant therapy (Fig. 2a). There was significant heterogeneity between the studies (*I*^*2*^ = 80 %, *P* < 0.001).

Six studies, comprising 1842 patients, were included in the meta-analysis investigating the effect of HR gain on OS [5,14,26,28,29,31]. There was no significant association found between HR gain and OS (HR 0.54 (95 % c.i. 0.15 to 1.87); *P* = 0.33) (Fig. 2b). Again, there was considerable heterogeneity between the studies (*I*^*2*^ = 90 %, *P* < 0.001).

### 3.4 Prognostic value of HR loss

**Fig. 3:**
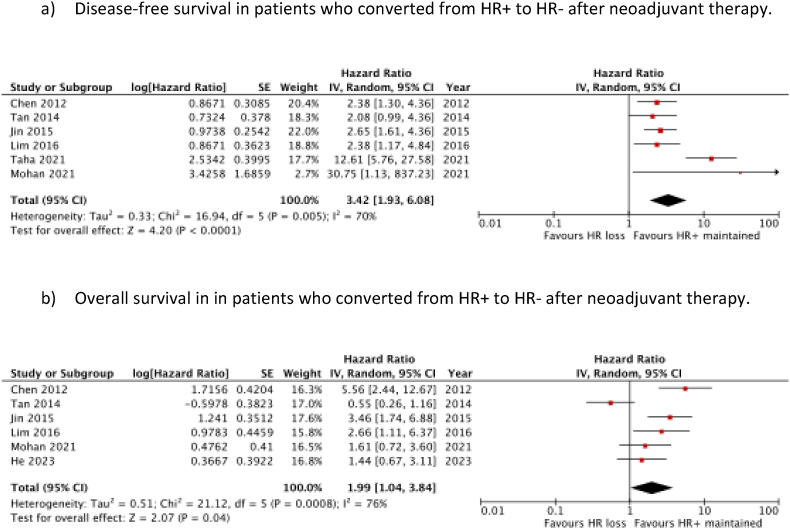
Prognostic significance of HR loss after neoadjuvant therapy.

A total of 6 studies evaluated the prognostic impact of HR loss on DFS [14,[28], [29], [30], [31], [32]]. Meta-analysis of these studies, consisting of 1462 patients, showed a significant benefit in DFS for patients who maintained their HR positivity, compared to those who lost HR positivity after neoadjuvant therapy (HR 3.42 (95 % c.i. 1.93 to 6.08); *P* < 0.001) (Fig. 3a). Meta-analysis of the 6 studies which investigated the effect of HR loss on OS [5,14,28,29,31,32] also found a significant benefit of remaining HR + after neoadjuvant therapy, compared to those who converted to HR- (HR 1.99 (95 % c.i. 1.04 to 3.84); *P* = 0.04) (Fig. 3b). Considerable heterogeneity existed between the studies (*I*^*2*^ = 76 %, *P* < 0.001).

### 3.5 Prognostic value of HER2 gain

**Fig. 4:**
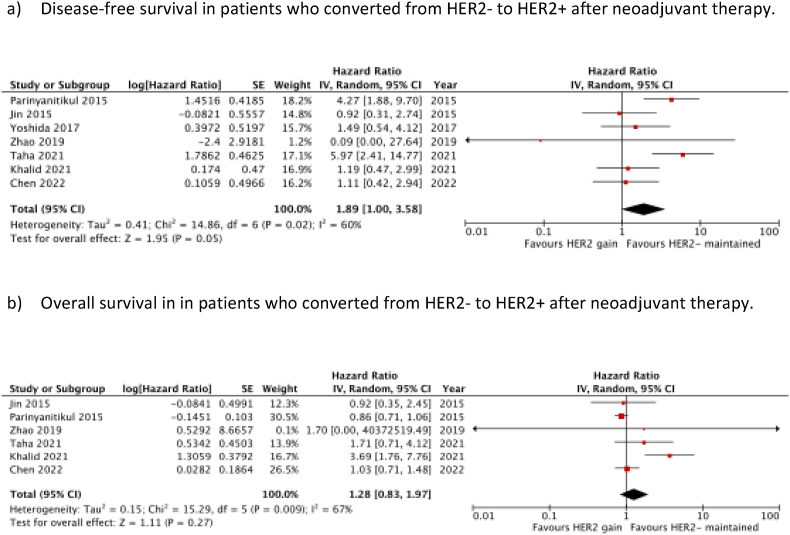
Prognostic significance of HER2 Gain after neoadjuvant therapy.

Seven studies were included in the analysis to determine the prognostic effect of HER2 gain after neoadjuvant therapy on DFS [14,27,30,[33], [34], [35], [36]]. Meta-analysis of these studies, including 2399 patients, demonstrated a significant DFS benefit in patients who remained HER2-compared to those who converted to HER2+ (HR 1.89 (95 % c.i. 1.00 to 3.58); *P* = 0.05) (Fig. 4a). However, on meta-analysis of 6 studies there was no significant association between HER2 gain and OS (HR 1.28 (95 % c.i. 0.83 to 1.97); *P* = 0.27) (Fig. 4b).

### 3.6 Prognostic value of HER2 loss

**Fig. 5:**
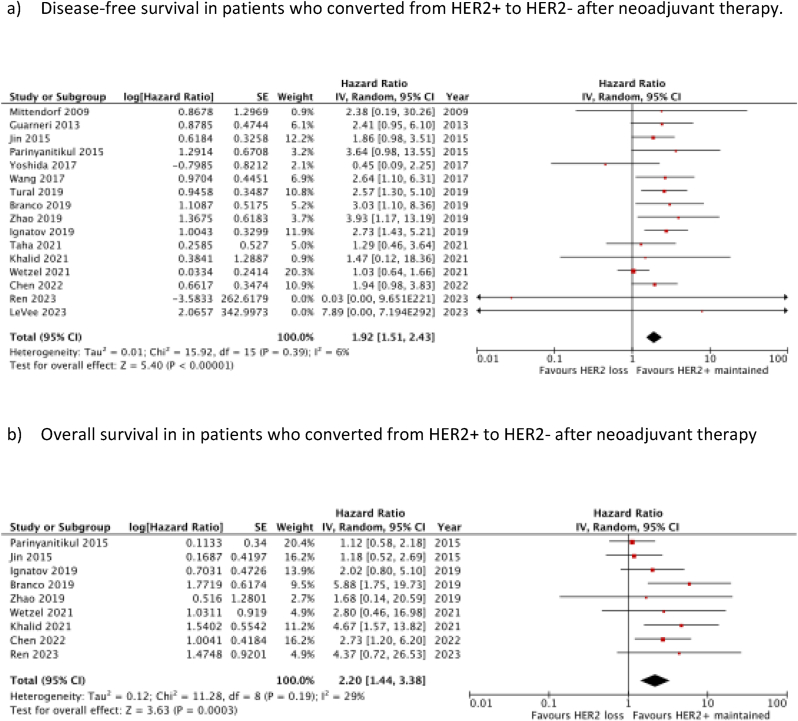
Prognostic significance of HER2 Loss after neoadjuvant therapy.

A total of 16 studies, comprising of 3951 patients, were included in the meta-analysis evaluating the prognostic value of HER2 loss on DFS. This found a significant association between improved DFS and remaining HER2+, compared to losing HER2 positivity after neoadjuvant therapy (HR 1.92 (95 % c.i. 1.51 to 2.43); *P* < 0.001) (Fig. 5a). A total of 9 studies investigated the effect of HER2 loss on OS (2608 patients). Meta-analysis of these also found a significant association between improved OS and remaining HER2+, compared with converting to HER2-after neoadjuvant treatment (HR 2.20 (95 % c.i. 1.44 to 3.38); *P* < 0.001) (Fig. 5b).

## 4 Discussion

This is by far the largest meta-analysis investigating the prognostic significance of receptor conversion after neoadjuvant therapy in breast cancer. As the number of patients undergoing neoadjuvant treatment has dramatically increased, there is considerable demand to determine accurate prognostic markers to inform treatment decisions. Receptor conversion after neoadjuvant therapy between the primary tumour and residual disease is common, with the potential to affect targeted treatment regimens. While there is substantial evidence of this phenomenon, the impact on long-term outcomes is less well established.

The present meta-analysis demonstrates that receptor conversion significantly impacts survival outcomes. Furthermore, it shows that the direction of change, rather than discordance alone, has the greatest effect on outcomes. This study found that a gain in HR after neoadjuvant therapy significantly improved DFS. The mechanism behind this conversion from HR-to HR + isn't fully understood, but a number of theories exist in the literature. It is well established that breast tumours are heterogenous, with both HR- and HR + cells potentially co-existing in the same tumour [37,38]. HR-cells are more chemosensitive, which could lead to selection pressure, with HR-cells being eliminated, leaving behind a higher proportion of HR + cells [39,40]. Another possibility is that chemotherapy induces epigenetic or gene expression changes, leading to the expression of hormone receptors in previously HR-cells [41,42]. Specifically in HER2+ breast cancer, there is increasing evidence that anti-HER2 therapy alone increases ER expression through crosstalk between these receptors [43]. A recently published study reported that this increase in ER expression after anti-HER2 therapy resulted in improved DFS, consistent with our findings [44]. This survival benefit may be due to the favourable biology of HR + tumours, or due to the possibility of adding endocrine therapy to their treatment regimens.

Conversely, a loss of HR positivity after neoadjuvant treatment was associated with significantly worse DFS and OS. These findings are consistent with the results of another meta-analysis, which similarly reported that conversion from HR + to HR-resulted in worse survival [15]. This is likely due to several factors. Firstly, endocrine therapies, such as tamoxifen or aromatase inhibitors, which target HR + cells, may be less effective at reducing recurrence and improving survival [45]. Second, this conversion may indicate a shift towards a more aggressive tumour phenotype, as HR-tumours are generally associated with worse survival outcomes [46]. Although not routinely recommended, neoadjuvant endocrine therapy is increasingly being used, particularly in postmenopausal women [47]. The literature on this remains limited, with potential implications on receptor conversion and survival still not well established [48].

While being HR + after neoadjuvant treatment, either remaining positive or converting, was associated with favourable survival outcomes in this study, the issue of HER2 conversion is more complex. This is highlighted by the results of the present study, which demonstrate that changing of HER2 status, regardless of direction, is associated with a worse prognosis. HER2-patients who remained negative after neoadjuvant therapy had significantly better DFS compared to those who gained HER2 positivity. This may be due to the fact that HER2+ disease is traditionally associated with higher proliferation rates and increased metastatic potential [49]. These tumours may also be adapting to therapy pressure, undergoing clonal selection, or the gain in HER2 expression may be coinciding with mutations in pathways such as PI3K/AKT, all of which could contribute to a more aggressive clinical course [[50], [51], [52]]. It is important to note that the KATHERINE trial demonstrated a significant survival benefit on the addition of adjuvant T-DM1 in patients with initially HER2+ breast cancer with residual disease [4]. Therefore, patients who gained HER2 positivity did not undergo neoadjuvant anti-HER2 therapy and were not eligible to receive adjuvant T-DM1, despite have HER2+ residual disease [4]. This highlights that HER2-patients who do not undergo retesting of their residual disease could have additional therapeutic options omitted, potentially worsening outcomes.

Interestingly, this meta-analysis demonstrates that conversion from HER2+ to HER2-is also associated with significantly worse DFS and OS than patients who remained HER2+ after neoadjuvant therapy. One theory is that neoadjuvant therapy, and anti-HER2 agents in particular, may select for HER2-negative clones that are more resistant or have different genetic alterations, making them more aggressive [53]. Another potential reason may be due to the decreased efficacy of HER2-directed therapies, which have proven to be effective in improving survival in HER2+ patients in the adjuvant setting [4]. Although adjuvant T-DM1 was effective even in HER2+ patients with HER2-residual disease, so this alone does not fully explain this [6]. Interestingly, while T-DM1 is still the mainstay of treatment in residual disease after neoadjuvant therapy in HER2+ breast cancer, the DESTINY-Breast05 trial is investigating whether a novel antibody-drug conjugate, trastuzumab deruxtecan (T-DXd), may have superior survival outcomes [54]. Given that T-DXd significantly outperformed T-DM1 in the metastatic setting [55,56], it is likely that it may do so in the residual disease setting also. A recent study found that in metastatic patients treated with T-DXd, HER2 positivity was lost in 32.4 % of patients [57]. This is significant given that T-DXd is currently also being investigated in the neoadjuvant setting in the DESTINY-Breast11 and SHAMROCK trials, with the potential for considerable HER2 loss in residual disease [58,59].

Given the dynamic nature of breast cancer biology, particularly in response to neoadjuvant therapy, there is an increasing need to refine post-treatment assessment and adapt therapeutic strategies accordingly. While retrospective data consistently show that changes in HR and HER2 status significantly impact prognosis, current guidelines do not adequately address how to manage these patients [14,29]. Liquid biopsy techniques, including circulating tumour cells (CTCs) and circulating tumour DNA (ctDNA), offer a promising complement to traditional histopathological evaluation of residual disease. These minimally-invasive tools allow for real-time monitoring of tumour heterogeneity and clonal evolution, which may not be captured in the surgical samples alone. Several studies have demonstrated the ability of CTCs to reveal HER2 and HR heterogeneity, and of ctDNA to detect emergent mutations and track treatment response [[60], [61], [62]]. Their integration into the clinic could the enhance detection of biologically significant receptor shifts and guide more tailored adjuvant therapy. There is also a critical need for prospective clinical trials that specifically address patients who experience receptor conversion after neoadjuvant therapy. Currently, despite increasing evidence that receptor conversion influences prognosis, therapeutic strategies for these patients remain largely focused on their baseline receptor status. Future trials should consider stratifying patients based on receptor conversion and incorporating adaptive treatment arms. Embedding liquid biopsy within these designs could enable early detection of conversion and real-time treatment adjustments. As receptor conversion is likely to become an increasingly recognised clinical phenomenon, these steps are essential to advancing personalised therapy and improving outcomes in this population.

The present systematic review and meta-analysis has several limitations. The majority of the studies included were retrospective in nature, rendering the data subject to the inherent limitations of incomplete data and potential confounders. There was heterogeneity in the molecular subtypes included, with most studies including all breast cancers and others including only specific molecular subtypes. Neoadjuvant treatment regimens and the use of targeted therapies also varied between studies as a result of this. There was also limited information on whether adjuvant treatment regimens were adjusted based on retesting results of residual disease. Although most studies reported overall use of endocrine and anti-HER2 therapies, few stratified these by post-treatment receptor status, limiting our ability to assess how receptor conversion influenced therapeutic decisions in clinical practice. Additionally, this study used aggregated rather than patient-level data. Another potential source of heterogeneity lies in the methodological differences in data extraction between studies, with some reporting HRs while others were extracted from survival curves.

In conclusion, this systematic review and meta-analysis demonstrates that receptor conversion following neoadjuvant therapy in breast cancer significantly impacts survival outcomes. Specifically, gaining HR positivity is associated with improved DFS, while losing HR positivity correlates with worse DFS and OS. With regards to HER2, gaining positivity is associated with worse DFS, and losing positivity is associated with worse DFS and OS, compared to patients who maintain their initial status. These findings underscore the importance of reassessing receptor status after neoadjuvant therapy to tailor subsequent treatment decisions accurately. Further research is needed to understand the mechanisms behind these conversions and to develop evidence-based guidelines for managing these patients in the era of personalised medicine, particularly given the evolving therapeutic landscape and the potential need for standardized protocols for routine retesting.

## CRediT authorship contribution statement

**Gavin P. Dowling:** Writing – review & editing, Writing – original draft, Project administration, Methodology, Investigation, Funding acquisition, Formal analysis, Data curation, Conceptualization. **Gordon R. Daly:** Writing – review & editing, Data curation. **Cian M. Hehir:** Writing – review & editing, Data curation. **Maen M. AlRawasdeh:** Writing – review & editing, Methodology, Investigation, Data curation. **Gavin G. Calpin:** Writing – review & editing, Formal analysis, Data curation. **Sami Almasri:** Writing – review & editing, Data curation. **Sinead Toomey:** Writing – review & editing, Supervision. **Leonie S. Young:** Writing – review & editing, Supervision, Conceptualization. **Bryan T. Hennessey:** Writing – review & editing, Supervision. **Arnold D.K. Hill:** Writing – review & editing, Supervision, Conceptualization.

## Ethics approval and consent to participate

Not applicable.

## Consent for publication

Not applicable.

## Availability of data and materials

The datasets used and/or analyzed during the current study are available from the corresponding author on reasonable request.

## Funding

This research was supported by the Royal College of Surgeons (10.13039/100012921RCSI)-Bon Secours Hospital Dublin StAR programme (grant number: 22252A01) and the 10.13039/501100022610Breast Cancer Ireland Avril Watters PhD scholarship (grant number: 24357A001).

## Declaration of competing interest

The authors have no conflicts of interest to declare.

## Abbreviations

ASCO: American Society of Clinical Oncology
CI: Confidence interval
DFS –: Disease-free survival
ESMO: European Society of Medical Oncology
HER2: Human epidermal growth factor receptor-2
HR: Hormone receptor
TNBC: Triple-negative breast cancer
MeSH: Medical Subject Heading
NCCN: National Comprehensive Cancer Network
OS –: Overall survival
T-DM1: Trastuzumab-emtansine
T-DXd –: Trastuzumab-deruxtecan

## References

[1] Cosgrove N., Eustace A.J., O'Donovan P. (2023). Predictive modelling of response to neoadjuvant therapy in HER2+ breast cancer. NPJ Breast Cancer..

[2] Dowling G.P., Keelan S., Toomey S., Daly G.R., Hennessy B.T., Hill A.D.K. (2023). Review of the status of neoadjuvant therapy in HER2-positive breast cancer. Front Oncol.

[3] Abdel-Razeq H., Khalil H., Assi H.I., Dargham T.B. (2022). Treatment strategies for residual disease following neoadjuvant chemotherapy in patients with early-stage breast cancer. Curr Oncol.

[4] Geyer C.E., Untch M., Huang C.-S. (2025). Survival with trastuzumab emtansine in residual HER2-positive breast cancer. N Engl J Med.

[5] He Y., Zhang J., Chen H. (2022). Clinical significance and prognostic value of receptor conversion after neoadjuvant chemotherapy in breast cancer patients. Front Surg.

[6] Loibl S., Huang C.S., Mano M.S. (2022). Adjuvant trastuzumab emtansine in HER2-positive breast cancer patients with HER2-negative residual invasive disease in KATHERINE. NPJ Breast Cancer.

[7] Cardoso F., Kyriakides S., Ohno S. (2019). Early breast cancer: ESMO Clinical Practice Guidelines for diagnosis, treatment and follow-up^†^. Ann Oncol.

[8] Korde L.A., Somerfield M.R., Carey L.A. (2021). Neoadjuvant chemotherapy, endocrine therapy, and targeted therapy for breast cancer: ASCO guideline. J Clin Oncol.

[9] Gradishar W.J., Moran M.S., Abraham J. (2024). Breast cancer, version 3.2024, NCCN clinical practice guidelines in Oncology. J Natl Compr Cancer Netw.

[10] Viale G., Fusco N. (2022). Pathology after neoadjuvant treatment – how to assess residual disease. Breast (Edinb).

[11] Vemuru S., Huang J., Colborn K. (2023). Clinical implications of receptor conversions in breast cancer patients who have undergone neoadjuvant chemotherapy. Breast Cancer Res Treat.

[12] Shaaban A.M., Provenzano E. (2022). Receptor status after neoadjuvant therapy of breast cancer: significance and implications. Pathobiology.

[13] Özdemir Ö., Zengel B., Kocatepe Çavdar D., Yılmaz C., Durusoy R. (2022). Prognostic value of receptor change after neoadjuvant chemotherapy in breast cancer patients. Eur J Breast Health.

[14] Jin X., Jiang Y.Z., Chen S., Yu K.D., Shao Z.M., Di G.H. (2015). Prognostic value of receptor conversion after neoadjuvant chemotherapy in breast cancer patients: a prospective observational study. Oncotarget.

[15] Li C., Fan H., Xiang Q. (2019). Prognostic value of receptor status conversion following neoadjuvant chemotherapy in breast cancer patients: a systematic review and meta-analysis. Breast Cancer Res Treat.

[16] Page M.J., McKenzie J.E., Bossuyt P.M. (2021). The PRISMA 2020 statement: an updated guideline for reporting systematic reviews. BMJ.

[17] Brooke B.S., Schwartz T.A., Pawlik T.M. (2021). MOOSE reporting guidelines for meta-analyses of observational studies. JAMA Surg.

[18] Schiavo J.H. (2019). PROSPERO: an international register of systematic review protocols. Med Ref Serv Q.

[19] von Elm E., Altman D.G., Egger M., Pocock S.J., Gøtzsche P.C., Vandenbroucke J.P. (2007). The Strengthening the Reporting of Observational Studies in Epidemiology (STROBE) statement: guidelines for reporting observational studies. Lancet.

[20] Stang A. (2010). Critical evaluation of the Newcastle-Ottawa scale for the assessment of the quality of nonrandomized studies in meta-analyses. Eur J Epidemiol.

[21] Borenstein M., Hedges L.V., Higgins J.P.T., Rothstein H.R. (2009).

[22] Tierney J.F., Stewart L.A., Ghersi D., Burdett S., Sydes M.R. (2007). Practical methods for incorporating summary time-to-event data into meta-analysis. Trials.

[23] *WebPlotDigitizer* [computer program]. Version 5.1: Automeris LLC..

[24] Drevon D., Fursa S.R., Malcolm A.L. (2017). Intercoder reliability and validity of WebPlotDigitizer in extracting graphed data. Behav Modif.

[25] Riley R.D., Higgins J.P.T., Deeks J.J. (2011). Interpretation of random effects meta-analyses. BMJ.

[26] Tacca O., Penault-Llorca F., Abrial C. (2007). Changes in and prognostic value of hormone receptor status in a series of operable breast cancer patients treated with neoadjuvant chemotherapy. Oncologist.

[27] Parinyanitikul N., Lei X., Chavez-Macgregor M. (2015). Receptor status change from primary to residual breast cancer after neoadjuvant chemotherapy and analysis of survival outcomes. Clin Breast Cancer.

[28] Tan Q.X., Qin Q.H., Yang W.P., Lian B., Wei C.Y. (2014). Prognostic value of hormone receptor status conversion following neoadjuvant chemotherapy in a series of operable breast cancer patients. Int J Clin Exp Pathol.

[29] Lim S.K., Lee M.H., Park I.H. (2016). Impact of molecular subtype conversion of breast cancers after neoadjuvant chemotherapy on clinical outcome. Cancer Res Treat.

[30] Taha H.F., Elfarargy O.M., Salem R.A., Mandour D., Salem A.A., Riad M., Concordance between Er P.R. (2021). HER2 neu receptors before and after neoadjuvant chemotherapy in locally advanced breast cancer. Forum Clin Oncol.

[31] Mohan S.C., Walcott-Sapp S., Lee M.K. (2021). Alterations in breast cancer biomarkers following neoadjuvant therapy. Ann Surg Oncol.

[32] Chen S., Chen C.M., Yu K.D., Zhou R.J., Shao Z.M. (2012). Prognostic value of a positive-to-negative change in hormone receptor status after neoadjuvant chemotherapy in patients with hormone receptor-positive breast cancer. Ann Surg Oncol.

[33] Yoshida A., Hayashi N., Suzuki K., Takimoto M., Nakamura S., Yamauchi H. (2017). Change in HER2 status after neoadjuvant chemotherapy and the prognostic impact in patients with primary breast cancer. J Surg Oncol.

[34] Zhao Y., Wang X., Huang Y., Zhou X., Zhang D. (2019). Conversion of immunohistochemical markers and breast density are associated with pathological response and prognosis in very young breast cancer patients who fail to achieve a pathological complete response after neoadjuvant chemotherapy. Cancer Manag Res.

[35] Al-Saleh K., Aldiab A., Salah T. (2021). Prognostic significance of HER2 expression changes following neoadjuvant chemotherapy in Saudi patients with locally advanced breast cancer. Clin Breast Cancer.

[36] Chen Y., Liu X., Yu K. (2022). Impact of hormone receptor, HER2, and Ki-67 status conversions on survival after neoadjuvant chemotherapy in breast cancer patients: a retrospective study. Ann Transl Med.

[37] Allott E.H., Geradts J., Sun X. (2016). Intratumoral heterogeneity as a source of discordance in breast cancer biomarker classification. Breast Cancer Res.

[38] Annaratone L., Simonetti M., Wernersson E. (2017). Quantification of HER2 and estrogen receptor heterogeneity in breast cancer by single-molecule RNA fluorescence in situ hybridization. Oncotarget.

[39] Zattarin E., Leporati R., Ligorio F. (2020). Hormone receptor loss in breast cancer: molecular mechanisms, clinical settings, and therapeutic implications. Cells.

[40] Teruya N., Inoue H., Horii R. (2023). Intratumoral heterogeneity, treatment response, and survival outcome of ER-positive HER2-positive breast cancer. Cancer Med.

[41] Galli G., Bregni G., Cavalieri S. (2017). Neoadjuvant chemotherapy exerts selection pressure towards luminal phenotype breast cancer. Breast Care.

[42] Tabuchi Y., Matsuoka J., Gunduz M. (2009). Resistance to paclitaxel therapy is related with Bcl-2 expression through an estrogen receptor mediated pathway in breast cancer. Int J Oncol.

[43] Giuliano M., Hu H., Wang Y.C. (2015). Upregulation of ER signaling as an adaptive mechanism of cell survival in HER2-positive breast tumors treated with anti-HER2 therapy. Clin Cancer Res.

[44] Dowling G.P., Daly G.R., Hegarty A. (2025). Neoadjuvant HER2 inhibition induces ESR1 DNA methylation alterations resulting in clinically relevant ER expression changes in breast cancers. Cancer Commun.

[45] Early Breast Cancer Trialists' Collaborative G (2011). Relevance of breast cancer hormone receptors and other factors to the efficacy of adjuvant tamoxifen: patient-level meta-analysis of randomised trials. Lancet.

[46] Bae S.Y., Kim S., Lee J.H. (2015). Poor prognosis of single hormone receptor- positive breast cancer: similar outcome as triple-negative breast cancer. BMC Cancer.

[47] Spring L.M., Gupta A., Reynolds K.L. (2016). Neoadjuvant endocrine therapy for estrogen receptor-positive breast cancer: a systematic review and meta-analysis. JAMA Oncol.

[48] Miligy I.M., Badr N., Stevens A. (2024). Pathological changes following neoadjuvant endocrine therapy (NAET): a multicentre study of 391 breast cancers. Int J Mol Sci.

[49] Slamon D.J., Clark G.M., Wong S.G., Levin W.J., Ullrich A., McGuire W.L. (1987). Human breast cancer: correlation of relapse and survival with amplification of the HER-2/neu oncogene. Science.

[50] Rasti A.R., Guimaraes-Young A., Datko F., Borges V.F., Aisner D.L., Shagisultanova E. (2022). PIK3CA mutations drive therapeutic resistance in human epidermal growth factor receptor 2–positive breast cancer. JCO Precis. Oncol..

[51] Chandarlapaty S., Sakr R.A., Giri D. (2012). Frequent mutational activation of the PI3K-AKT pathway in trastuzumab-resistant breast cancer. Clin Cancer Res.

[52] Dowling G.P., Keelan S., Cosgrove N.S. (2024). Receptor Discordance in Metastatic Breast Cancer; a review of clinical and genetic subtype alterations from primary to metastatic disease. Breast Cancer Res Treat.

[53] Morganti S., Ivanova M., Ferraro E. (2022). Loss of HER2 in breast cancer: biological mechanisms and technical pitfalls. Cancer Drug Resis..

[54] A study of trastuzumab deruxtecan (T-DXd) versus trastuzumab emtansine (T-DM1) in high-risk HER2-positive participants with residual invasive breast cancer following neoadjuvant therapy (DESTINY-Breast05). https://clinicaltrials.gov/study/NCT04622319.

[55] Cortés J., Hurvitz S.A., Im S.-A. (2024). Trastuzumab deruxtecan versus trastuzumab emtansine in HER2-positive metastatic breast cancer: long-term survival analysis of the DESTINY-Breast03 trial. Nat Med.

[56] Dowling G.P., Daly G.R., Keelan S. (2023). Efficacy and safety of trastuzumab deruxtecan in breast cancer: a systematic review and meta-analysis. Clin Breast Cancer.

[57] Gouda M.A., Gonugunta A., Dumbrava E.E. (2025). Human epidermal growth factor receptor 2 loss following treatment with trastuzumab deruxtecan in patients with metastatic breast cancer. Clin Cancer Res.

[58] Trastuzumab deruxtecan (T-DXd) alone or in sequence with THP, versus standard treatment (ddAC-THP) HER2-positive early breast cancer. https://ClinicalTrials.gov/show/NCT05113251.

[59] Dowling G.P., Toomey S., Bredin P. (2024). Neoadjuvant trastuzumab deruxtecan (T-DXd) with response-directed definitive therapy in early stage HER2-positive breast cancer: a phase II study protocol (SHAMROCK study). BMC Cancer.

[60] Mazzitelli C., Santini D., Corradini A.G. (2023). Liquid biopsy in the management of breast cancer patients: where are we now and where are we going. Diagnostics.

[61] Huai J., Cao M., Jiang Y. (2021). [Retracted] evaluation of liquid biopsy in patients with HER2‐positive breast cancer. BioMed Res Int.

[62] Di Cosimo S., De Marco C., Silvestri M. (2023). Can we define breast cancer HER2 status by liquid biopsy?. Int. Rev. Cell. Mol. Biol..

[63] Mittendorf E.A., Wu Y., Scaltriti M. (2009). Loss of HER2 amplification following trastuzumab-based neoadjuvant systemic therapy and survival outcomes. Clin Cancer Res.

[64] Guarneri V., Dieci M.V., Barbieri E. (2013). Loss of HER2 positivity and prognosis after neoadjuvant therapy in HER2-positive breast cancer patients. Ann Oncol.

[65] Wang R.X., Chen S., Jin X., Chen C.M., Shao Z.M. (2017). Weekly paclitaxel plus carboplatin with or without trastuzumab as neoadjuvant chemotherapy for HER2-positive breast cancer: loss of HER2 amplification and its impact on response and prognosis. Breast Cancer Res Treat.

[66] Branco F.P., Machado D., Silva F.F. (2019). Loss of HER2 and disease prognosis after neoadjuvant treatment of HER2+ breast cancer. Am J Tourism Res.

[67] Ignatov T., Gorbunow F., Eggemann H., Ortmann O., Ignatov A. (2019). Loss of HER2 after HER2-targeted treatment. Breast Cancer Res Treat.

[68] Tural D., Karaca M., Zirtiloglu A., Hacioglu B.M., Sendur M.A.N., Ozet A. (2019). Receptor discordances in locally advanced breast cancer after neoadjuvant chemotherapy and their effects on survival. J. BUON.

[69] Wetzel C.L., Sutton T.L., Gardiner S., Farinola M., Johnson N., Garreau J.R. (2021). Loss of HER2-positivity following neoadjuvant targeted therapy for breast cancer is not associated with inferior oncologic outcomes. J Surg Oncol.

[70] LeVee A., Spector K., Larkin B. (2023). Incidence and prognostic impact of HER2-positivity loss after dual HER2-directed neoadjuvant therapy for HER2+ breast cancer. Cancer Med.

[71] Ren X., Zhang X., Ma X. (2023). Changes in HER2 status and survival outcomes in patients with non-pathological complete response after neoadjuvant targeted treatment. Medicine (Baltim).

