# Supplementary material for: Prognostic significance of receptor conversion following neoadjuvant therapy in breast cancer: a systematic review & meta-analysis
Source: Breast. 2025 Jun 9;82:104516. doi: 10.1016/j.breast.2025.104516 (PMC12198027; doi:10.1016/j.breast.2025.104516)
Supplement: Multimedia component 1 [file mmc1.docx]

| Author | Year | STROBE Score (n/22) |
| --- | --- | --- |
| Tacca^1^ | 2007 | 20 |
| Mittendorf^2^ | 2009 | 20 |
| Chen^3^ | 2012 | 20 |
| Guarneri^4^ | 2013 | 20 |
| Tan^5^ | 2014 | 20 |
| Jin^6^ | 2015 | 20 |
| Parinyanitikul^7^ | 2015 | 21 |
| Lim^8^ | 2016 | 22 |
| Wang^9^ | 2017 | 20 |
| Yoshida^10^ | 2017 | 21 |
| Branco^11^ | 2019 | 20 |
| Ignatov^12^ | 2019 | 21 |
| Tural^13^ | 2019 | 20 |
| Zhao^14^ | 2019 | 20 |
| Khalid Al-Saleh^15^ | 2021 | 21 |
| Mohan^16^ | 2021 | 21 |
| Taha^17^ | 2021 | 22 |
| Wetzel^18^ | 2021 | 21 |
| Chen^19^ | 2022 | 21 |
| He^20^ | 2023 | 21 |
| LeVee^21^ | 2023 | 22 |
| Ren^22^ | 2023 | 20 |

Table S1. STROBE (Strengthening The Reporting of Observational Studies in Epidemiology) Statement checklist scores for included studies.

| Author | Year | STROBE Score (n/22) |
| --- | --- | --- |
| Tacca^1^ | 2007 | 20 |
| Mittendorf^2^ | 2009 | 20 |
| Chen^3^ | 2012 | 20 |
| Guarneri^4^ | 2013 | 20 |
| Tan^5^ | 2014 | 20 |
| Jin^6^ | 2015 | 20 |
| Parinyanitikul^7^ | 2015 | 21 |
| Lim^8^ | 2016 | 22 |
| Wang^9^ | 2017 | 20 |
| Yoshida^10^ | 2017 | 21 |
| Branco^11^ | 2019 | 20 |
| Ignatov^12^ | 2019 | 21 |
| Tural^13^ | 2019 | 20 |
| Zhao^14^ | 2019 | 20 |
| Khalid Al-Saleh^15^ | 2021 | 21 |
| Mohan^16^ | 2021 | 21 |
| Taha^17^ | 2021 | 22 |
| Wetzel^18^ | 2021 | 21 |
| Chen^19^ | 2022 | 21 |
| He^20^ | 2023 | 21 |
| LeVee^21^ | 2023 | 22 |
| Ren^22^ | 2023 | 20 |

Table S2. Newcastle-Ottawa Scale (NOS) scores for assessing the quality of included studies.

| Author | Year | NOS Score (n/9) |
| --- | --- | --- |
| Tacca^1^ | 2007 | 7 |
| Mittendorf^2^ | 2009 | 7 |
| Chen^3^ | 2012 | 9 |
| Guarneri^4^ | 2013 | 6 |
| Tan^5^ | 2014 | 7 |
| Jin^6^ | 2015 | 7 |
| Parinyanitikul^7^ | 2015 | 9 |
| Lim^8^ | 2016 | 7 |
| Wang^9^ | 2017 | 7 |
| Yoshida^10^ | 2017 | 6 |
| Branco^11^ | 2019 | 6 |
| Ignatov^12^ | 2019 | 6 |
| Tural^13^ | 2019 | 6 |
| Zhao^14^ | 2019 | 6 |
| Khalid Al-Saleh^15^ | 2021 | 6 |
| Mohan^16^ | 2021 | 9 |
| Taha^17^ | 2021 | 7 |
| Wetzel^18^ | 2021 | 7 |
| Chen^19^ | 2022 | 9 |
| He^20^ | 2023 | 9 |
| LeVee^21^ | 2023 | 6 |
| Ren^22^ | 2023 | 6 |

Table S3. Cut-off values of tumour nuclei staining for ER/PR on IHC used to define hormone receptor positivity in included studies.

| Author | Year | % staining positivity |
| --- | --- | --- |
| Tacca^1^ | 2007 | ≥10% |
| Mittendorf^2^ | 2009 | NR |
| Chen^3^ | 2012 | ≥1% |
| Guarneri^4^ | 2013 | NR |
| Tan^5^ | 2014 | ≥1% |
| Jin^6^ | 2015 | ≥1% |
| Parinyanitikul^7^ | 2015 | ≥5% |
| Lim^8^ | 2016 | NR |
| Wang^9^ | 2017 | ≥1% |
| Yoshida^10^ | 2017 | NR |
| Branco^11^ | 2019 | NR |
| Ignatov^12^ | 2019 | NR |
| Tural^13^ | 2019 | ≥1% |
| Zhao^14^ | 2019 | ≥1% |
| Khalid Al-Saleh^15^ | 2021 | ≥1% |
| Mohan^16^ | 2021 | NR |
| Taha^17^ | 2021 | NR |
| Wetzel^18^ | 2021 | NR |
| Chen^19^ | 2022 | NR |
| He^20^ | 2023 | ≥1% |
| LeVee^21^ | 2023 | NR |
| Ren^22^ | 2023 | NR |

NR; not reported

**Figure S1.** Prognostic significance of HR gain after neoadjuvant therapy: Subgroups divided based on method of HR extraction.

1. Disease-free survival in patients who converted from HR- to HR+ after neoadjuvant therapy.
2. Overall survival in in patients who converted from HR- to HR+ after neoadjuvant therapy.

**Figure S2.** Prognostic significance of HR loss after neoadjuvant therapy: Subgroups divided based on method of HR extraction.

1. Disease-free survival in patients who converted from HR+ to HR- after neoadjuvant therapy.

1. Overall survival in in patients who converted from HR+ to HR- after neoadjuvant therapy.

**Figure S3.** Prognostic significance of HER2 Gain after neoadjuvant therapy: Subgroups divided based on method of HR extraction.

1. Disease-free survival in patients who converted from HER2- to HER2+ after neoadjuvant therapy.

1. Overall survival in in patients who converted from HER2- to HER2+ after neoadjuvant therapy.

**Figure S4.** Prognostic significance of HER2 Loss after neoadjuvant therapy: Subgroups divided based on method of HR extraction.

1. Disease-free survival in patients who converted from HER2+ to HER2- after neoadjuvant therapy.

1. Overall survival in in patients who converted from HER2+ to HER2- after neoadjuvant therapy

**Appendix S1 Search Strategy**

PubMed:

breast neoplasm*[MESH] OR "breast cancer*"[Text Word] OR "breast tumour*"[Text Word] OR

"breast tumor*"[Text Word] OR "mammary neoplasm*"[Text Word] OR "mammary cancer*"[Text Word] OR "breast carcinoma*"[Text Word]

AND

Neoadjuvant therapy*[MESH] OR “neoadjuvant treatment*”[Text word] OR “neoadjuvant

chemotherapy*”[Text word]

AND

Receptor, ErbB-2*[MESH] OR Triple Negative Breast Neoplasms*[MESH] OR Receptors,

Progesterone*[MESH] OR Receptors, Estrogen*[MESH] OR “receptor*”[All fields] OR “molecular subtype”[tiab:~0]

AND

“change*”[All fields] OR “discordance*”[All fields] OR “switch*”[All fields]

Embase:

'breast disease'/exp OR 'breast disease' OR 'breast cancer'/exp OR 'breast cancer' OR 'breast

tumor'/exp OR 'breast tumor'

AND

'neoadjuvant therapy'/exp OR 'neoadjuvant therapy' OR ‘neoadjuvant chemotherapy’/exp

AND

‘receptor’:ti,ab,kw OR 'molecular subtype':ti,ab,kw OR 'epidermal growth factor receptor 2'/exp OR 'triple negative breast cancer'/exp OR 'estrogen receptor'/exp OR 'estrogen receptor positive breast cancer'/exp OR 'progesterone receptor'/exp OR 'progesterone receptor positive breast cancer'/exp

AND

'change':ti,ab,kw OR 'discordance':ti,ab,kw OR 'loss':ti,ab,kw OR 'gain':ti,ab,kw

Scopus:

TITLE-ABS-KEY ( 'breast AND neoplasm$' OR 'breast AND cancer$' OR 'breast AND carcinoma$' )

AND ALL

( 'neoadjuvant' OR 'neoadjuvant AND treatment' OR 'neoadjuvant AND chemotherapy' OR 'neoadjuv

ant AND therapy' ) AND ( TITLE-ABS-KEY ( receptor OR molecular AND subtype ) ) OR ( TITLE-ABS-KEY

( 'her2' OR 'human AND epidermal AND growth AND factor' OR 'triple AND negative' OR 'tnbc' OR 'es

trogen AND receptor' OR 'progesterone AND receptor' ) )

AND ALL

( change OR discordance OR switch OR loss OR gain )

The Cochrane Library:


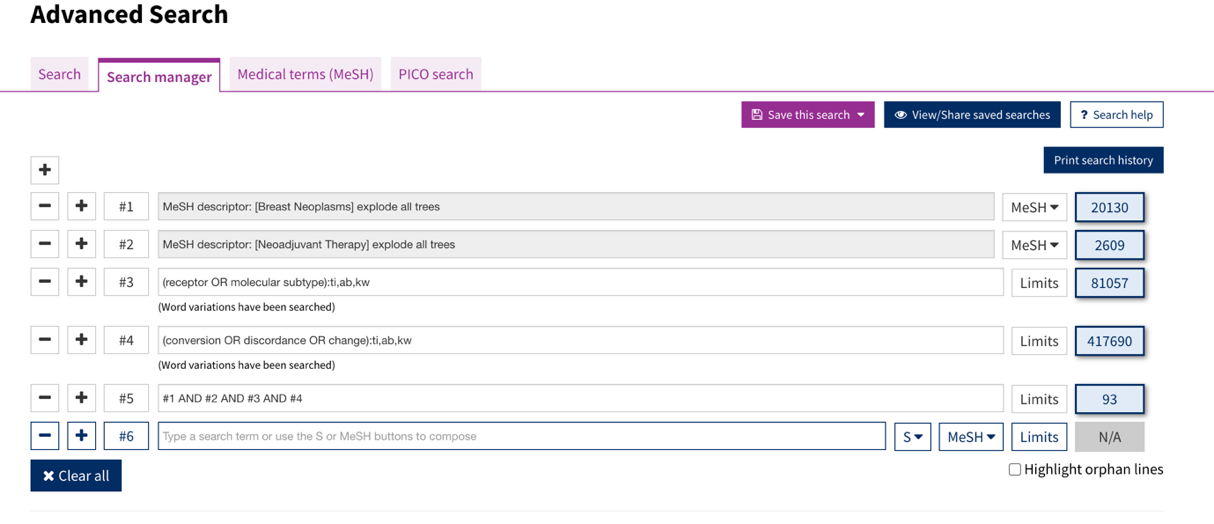


1. Tacca O, Penault-Llorca F, Abrial C, et al. Changes in and prognostic value of hormone receptor status in a series of operable breast cancer patients treated with neoadjuvant chemotherapy. *Oncologist.* 2007;12(6):636-643.

2. Mittendorf EA, Wu Y, Scaltriti M, et al. Loss of HER2 amplification following trastuzumab-based neoadjuvant systemic therapy and survival outcomes. *Clinical Cancer Research.* 2009;15(23):7381-7388.

3. Chen S, Chen CM, Yu KD, Zhou RJ, Shao ZM. Prognostic value of a positive-to-negative change in hormone receptor status after neoadjuvant chemotherapy in patients with hormone receptor-positive breast cancer. *Annals of Surgical Oncology.* 2012;19(9):3002-3011.

4. Guarneri V, Dieci MV, Barbieri E, et al. Loss of HER2 positivity and prognosis after neoadjuvant therapy in HER2-positive breast cancer patients. *Annals of Oncology.* 2013;24(12):2990-2994.

5. Tan QX, Qin QH, Yang WP, Lian B, Wei CY. Prognostic value of hormone receptor status conversion following neoadjuvant chemotherapy in a series of operable breast cancer patients. *International journal of clinical and experimental pathology.* 2014;7(7):4086-4094.

6. Jin X, Jiang YZ, Chen S, Yu KD, Shao ZM, Di GH. Prognostic value of receptor conversion after neoadjuvant chemotherapy in breast cancer patients: a prospective observational study. *Oncotarget.* 2015;6(11):9600-9611.

7. Parinyanitikul N, Lei X, Chavez-Macgregor M, et al. Receptor status change from primary to residual breast cancer after neoadjuvant chemotherapy and analysis of survival outcomes. *Clinical Breast Cancer.* 2015;15(2):153-160.

8. Lim SK, Lee MH, Park IH, et al. Impact of Molecular Subtype Conversion of Breast Cancers after Neoadjuvant Chemotherapy on Clinical Outcome. *Cancer Res Treat.* 2016;48(1):133-141.

9. Wang RX, Chen S, Jin X, Chen CM, Shao ZM. Weekly paclitaxel plus carboplatin with or without trastuzumab as neoadjuvant chemotherapy for HER2-positive breast cancer: loss of HER2 amplification and its impact on response and prognosis. *Breast Cancer Research and Treatment.* 2017;161(2):259-267.

10. Yoshida A, Hayashi N, Suzuki K, Takimoto M, Nakamura S, Yamauchi H. Change in HER2 status after neoadjuvant chemotherapy and the prognostic impact in patients with primary breast cancer. *Journal of Surgical Oncology.* 2017;116(8):1021-1028.

11. Branco FP, Machado D, Silva FF, et al. Loss of HER2 and disease prognosis after neoadjuvant treatment of HER2+ breast cancer. *American Journal of Translational Research.* 2019;11(9):6110-6116.

12. Ignatov T, Gorbunow F, Eggemann H, Ortmann O, Ignatov A. Loss of HER2 after HER2-targeted treatment. *Breast Cancer Research and Treatment.* 2019;175(2):401-408.

13. Tural D, Karaca M, Zirtiloglu A, Hacioglu BM, Sendur MAN, Ozet A. Receptor discordances in locally advanced breast cancer after neoadjuvant chemotherapy and their effects on survival. *Journal of BUON.* 2019;24(1):20-25.

14. Zhao Y, Wang X, Huang Y, Zhou X, Zhang D. Conversion of immunohistochemical markers and breast density are associated with pathological response and prognosis in very young breast cancer patients who fail to achieve a pathological complete response after neoadjuvant chemotherapy. *Cancer Management and Research.* 2019;11:5677-5690.

15. Al-Saleh K, Aldiab A, Salah T, et al. Prognostic Significance of HER2 Expression Changes Following Neoadjuvant Chemotherapy in Saudi Patients With Locally Advanced Breast Cancer. *Clinical Breast Cancer.* 2021;21(4):e362-e367.

16. Mohan SC, Walcott-Sapp S, Lee MK, et al. Alterations in Breast Cancer Biomarkers Following Neoadjuvant Therapy. *Annals of Surgical Oncology.* 2021;28(11):5907-5917.

17. Taha HF, Elfarargy OM, Salem RA, Mandour D, Salem AA, Riad M. Concordance between ER, PR, HER2 neu Receptors before and after Neoadjuvant Chemotherapy in Locally Advanced Breast Cancer. *Forum of Clinical Oncology.* 2021;12(1):3-11.

18. Wetzel CL, Sutton TL, Gardiner S, Farinola M, Johnson N, Garreau JR. Loss of HER2-positivity following neoadjuvant targeted therapy for breast cancer is not associated with inferior oncologic outcomes. *Journal of Surgical Oncology.* 2021;124(8):1224-1234.

19. Chen Y, Liu X, Yu K, et al. Impact of hormone receptor, HER2, and Ki-67 status conversions on survival after neoadjuvant chemotherapy in breast cancer patients: a retrospective study. *Annals of Translational Medicine.* 2022;10(2).

20. He Y, Zhang J, Chen H, et al. Clinical significance and prognostic value of receptor conversion after neoadjuvant chemotherapy in breast cancer patients. *Front Surg.* 2022;9:1037215.

21. LeVee A, Spector K, Larkin B, et al. Incidence and prognostic impact of HER2-positivity loss after dual HER2-directed neoadjuvant therapy for HER2+ breast cancer. *Cancer Medicine.* 2023;12(9):10647-10659.

22. Ren X, Zhang X, Ma X, et al. Changes in HER2 status and survival outcomes in patients with non-pathological complete response after neoadjuvant targeted treatment. *Medicine (Baltimore).* 2023;102(39):e34903.
